# Ecto- and endoparasites of common reedbuck, *Redunca arundinum*, at 2 localities in KwaZulu-Natal Province, South Africa: community and network structure

**DOI:** 10.1017/S0031182024000532

**Published:** 2024-06

**Authors:** Kerstin Junker, Joop Boomker, Ivan G. Horak, Boris R. Krasnov

**Affiliations:** 1 National Collection of Animal Helminths, Epidemiology, Parasites and Vectors Programme, ARC-Onderstepoort Veterinary Institute, Onderstepoort 0110, South Africa; 2 Department of Veterinary Tropical Diseases, University of Pretoria, Onderstepoort 0110, South Africa; 3 Mitrani Department of Desert Ecology, Swiss Institute for Dryland Environmental and Energy Research, Jacob Blaustein Institutes for Desert Research, Ben-Gurion University of the Negev, 8499000 Midreshet Ben-Gurion, Israel

**Keywords:** helminths, host–parasite interactions, infracommunities, lice, nestedness, ticks

## Abstract

Parasite community structure is governed by functional traits of hosts and parasites. Notably, parasite populations and communities respond to host social and spatial behaviour. Many studies demonstrating these effects dealt with small-bodied host species, while the influence of host social patterns on parasite communities in large hosts remains understudied. In an earlier study on nyalas (*Tragelaphus angasii*), host age was more important than sex in structuring helminth communities and networks, but the influence of both was mediated by local environmental conditions, creating different locality patterns. Common reedbuck (*Redunca arundinum*) differ from nyalas in spatial and social behaviour. Based on helminth and ectoparasite data from 56 reedbuck examined at 2 localities in KwaZulu-Natal Province, we asked which patterns are similar and which differ between the 2 host species. Similar to nyalas, reedbuck age was more important than sex in structuring communities and networks. However, local environmental conditions exerted the strongest influence on transmission patterns, especially in ectoparasites. Complex interactions between reedbuck traits, parasite traits and local environmental conditions modulated the risk of infection differently at the 2 sites, confirming our earlier findings in nyalas that pooling data from different locations may obscure location-specific parasite community patterns. Similarities between patterns in reedbuck and nyalas, despite their behavioural differences, suggest some common patterns in parasite community ecology that, in turn, are determined mostly by parasite traits and population dynamics.

## Graphical abstract

**Figure:**
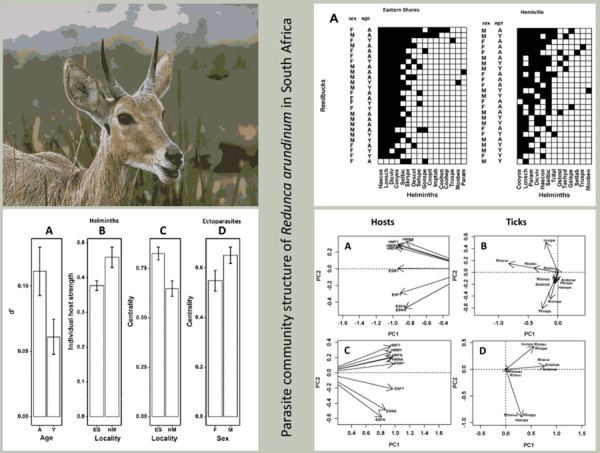

## Introduction

Studies on the patterns and processes that govern parasite community structure have shown that the interactions between host and parasite species are mediated by functional traits of both. From the aspect of the parasite, transmission success can be affected by, for example, its life cycle – direct or indirect, transmission mode, preferred site in the host, fecundity and/or host specificity (Cable *et al*., 2017; Cardoso *et al*., 2020, 2021). On the other hand, given the intimate ties between a host and its parasites, it is not surprising that host traits, morphological, physiological, behavioural or ecological, also affect parasite population and community ecology.

In particular, parasite populations and communities respond to host social structure and spatial behaviour. Effects of host social and spatial behaviour on the transmission of both ecto- and endoparasites have been demonstrated in a number of studies, and positive correlations between group living and/or host group size and infection parameters such as parasite abundance, prevalence or parasite species richness have been found in a variety of ectoparasites or helminths (Arneberg *et al*., 1998; Stanko *et al*., 2002; Altizer *et al*., 2003; Patterson and Ruckstuhl, 2013). However, many of these studies dealt with fish (Poulin, 1991; Luque *et al*., 2004), avian hosts (Brown and Brown, 1986; Moore *et al*., 1988) or rodents (Bordes *et al*., 2007; Spickett *et al*., 2017*a*). This obvious focus on small-bodied host species is likely because of the relative ease with which replicate samples can be obtained, whereas in large hosts, the influence of host social patterns on parasite communities is still poorly understood.

In African antelopes (Bovidae), social structure and spatial behaviour is highly variable. In order to study associations between host social organization and gastrointestinal parasite burdens, established on the basis of faecal egg counts, Ezenwa (2004) assigned African bovids to 4 categories, based on grouping behaviour and spatial distribution according to Estes (1974): solitary/territorial, solitary/non-territorial, gregarious/territorial and gregarious/non-territorial. In addition to this, intraspecific variation in gregarious species in group size and group stability as well as the existence of social classes (territorial males, bachelor males and nursery herd members) were found to result in intraspecific differences in parasite transmission and infection risk (Ezenwa, 2004).

In an earlier study on the helminth communities of nyalas (*Tragelaphus angasii*), at 3 localities in South Africa, we found that host age was an important determinant of parasite community structure, while host sex was of less importance (Junker *et al*., 2022). Notably, the effects of host sex and age were mitigated by environmental factors and thus varied at the 3 localities. Nyalas are polygamous and non-territorial. While typically occurring in small groups, they are considered a gregarious species, with groups being fluid in their composition (Skinner and Chimimba, 2005). Parasite transmission opportunities are higher among gregarious species (Ezenwa, 2004), and consequently, the patterns observed in nyalas may differ from those in solitary antelopes.

To compare findings in social/polygamous *vs* solitary/monogamous antelopes, we studied the effects of sex and age and their variation across space (2 localities) on patterns of parasite abundance, species richness, community structure and individual-based host–parasite networks in common reedbuck (*Redunca arundinum*). Reedbuck are not gregarious, occurring mainly singly or in pairs but also in small family groups of 2–3 individuals. Adult males tend to become solitary towards summer, and females are known to isolate themselves for about a month before and up to 4 months after parturition (Estes, 1974; Skinner and Chimimba, 2005). Female reedbuck are non-territorial, but males might display varying degrees of territoriality depending on locality (see below; Skinner and Chimimba, 2005). Following the reasoning of Ezenwa (2004) that higher levels of aggregation and associated transmission opportunities in gregarious host species should lead to higher parasite species richness when compared to solitary species, we expected to find helminth species richness to be lower in reedbuck than nyalas. Howard (1986) reported that of 566 different sightings of reedbuck, approximately 36% were solitary animals. These did not only include adult males and females, but also subadults that had separated from their nursery group. Given that a solitary lifestyle is typical for both males and females as well as adult and young animals, we expected the effect of age or sex to be less pronounced in reedbuck than nyalas.

In order to facilitate comparison between helminth communities from reedbuck and those from nyalas, we analysed the data on reedbuck endoparasites in the same way as we had done for nyalas. Since data on the ectoparasites of these reedbucks were also available, we looked at the effect of host sex and age as well as locality on the population and community ecology of ectoparasites and asked whether these effects are similar between endo- and ectoparasites. We predicted that effects would differ between the 2 groups because of a much stronger environmental effect on ectoparasites relative to endoparasites. Consequently, the effect of locality on ectoparasites is expected to exceed that of host sex and age.

## Material and methods

### Host species

Common reedbuck used to be widespread in South Africa, but loss of specialized habitat has reduced their geographic range. Today, they occur in the central parts of Limpopo Province, eastern Mpumalanga Province and the extreme east of North West Province. They are widely distributed in KwaZulu-Natal, especially on the eastern and western shores of Lake St Lucia in the isiMangaliso Wetland Park. Suitable habitat must meet 2 essential requirements: a water supply, and tall grass or reeds to provide cover as well as shelter against loss of body heat, especially during the harsh winters in the Drakensberg foothills (Skinner and Chimimba, 2005). While temporary groups of 15–20 animals may form briefly during cold and dry weather periods, reedbuck, as mentioned above, are not gregarious and live predominantly solitarily or in pairs. Small groups of 2–3 animals, including female groups, bachelor groups or family groups, have also been observed. Territoriality in males appears to vary depending on locality. Males in the highland regions of KwaZulu-Natal become territorial during rut, with dominant males defending territories closest to the food source. At St Lucia, a social hierarchy allows dominant males to mate with females in oestrus (Skinner and Chimimba, 2005). Common reedbuck are grazers but under exceptional circumstances, such as during winter when the quality of grasses is low, may supplement their diet with herbs or browse. Where available, they will readily use agriculturally irrigated pastures (Skinner and Chimimba, 2005).

### Study areas

Common reedbuck were collected from 2 localities in KwaZulu-Natal Province in South Africa (Horak *et al*., 1988; Boomker *et al*., 1989*b*). Animals examined at Himeville (HM) were collected from 5 adjacent farms in the vicinity (29°43’ S; 29°36’ E; 1550–2000 m above sea level). Himeville district is situated in a vegetation unit of the Grassland Biome called the Drakensberg Foothill Moist Grassland. The latter is characterized by forb-rich grassland dominated by short bunch grasses, including *Themeda triandra* and *Tristachya leucothrix* (Mucina and Rutherford, 2006). However, at the time of sample collection, artificial, often irrigated, annual permanent pastures, and croplands of maize and Japanese radish were also prominent landscape features (Horak *et al*., 1988). Himeville is part of the summer rainfall region with a mean annual precipitation of 890 mm. Most of the 1220 mm of rain recorded during the 13 months during which animals were collected fell between October and March. The mean annual temperature is 14.6°C; winters can be severe with an average of 26 frost days per year (Mucina and Rutherford, 2006).

Other antelope species occurring sympatrically with common reedbuck in the Himeville area were eland (*Taurotragus oryx*), mountain reedbuck (*Redunca fulvorufula*), grey rhebok (*Pelea capreolus*), oribi (*Ourebia ourebi*), blesbok (*Damaliscus dorcas phillipsi*) and common duiker (*Sylvicapra grimmia*). Farmed animals comprised mainly cattle and sheep (Horak *et al*., 1988).

At the time of sample collection, Eastern Shores Nature Reserve (27°51’ S to 28°25’ S; 32°20’ E to 32°40’ E; ca. 250 km^2^), Charters Creek Nature Reserve and St Lucia Game Park formed adjacent conservation areas in today's iSimangaliso Wetland Park (see Horak *et al*., 1988). These conservation areas, referred to as ESNR below, are situated within a patchwork of vegetation units of the Indian Ocean Coastal Belt, including the Maputaland Coastal Belt, the Maputaland Wooded Grassland and Subtropical Freshwater Wetlands (Mucina and Rutherford, 2006). Within these areas, the preferred habitat of reedbuck was low-lying, seasonally inundated grassland (Horak *et al*., 1988). Mean annual rainfall in the 3 vegetation units is 933–989 mm, but was 1109 mm at the time of sample collection, with January to April being the wettest and July to September the driest months. Mean annual temperature is 19.6–21.1°C, with the hottest month being February and the coolest July; winters are frost-free (Horak *et al*., 1988; Mucina and Rutherford, 2006).

Other mammal species present at ESNR at the time reedbuck were collected were hippopotamus (*Hippopotamus amphibius*), bushpig (*Potamochoerus porcus*), common warthog (*Phacochoerus africanus*), buffalo (*Syncerus caffer*), bushbuck (*Tragelaphus scriptus*), nyala, common duiker, steenbok (*Raphicerus campestris*), waterbuck (*Kobus ellipsiprymnus*), blue wildebeest (*Connochaetes taurinus*) and impala (*Aepyceros melampus*). Population densities of reedbuck were high, ranging from 0.46 to 0.86 per ha (Horak *et al*., 1988).

### Host and parasite collection

A total of 26 animals (4 adult and 10 young males; 9 adult and 3 young females) were collected from Himeville. Two reedbuck each, 1 adult and 1 young animal, were examined monthly from May 1983 to May 1984. Only helminth but no tick data are available for a single young male, collected in November 1983.

At ESNR, 30 reedbuck were examined (10 adult and 5 young males; 9 adult and 6 young females). Of these, 2–3 reedbuck were taken at 3-monthly intervals from March 1983 to April 1984 and an additional 5 and 4 animals were examined during August 1984 and January 1987, respectively. Males and females were classified as adult based on body size, pelage and/or horn conformation and length (Howard, 1983).

Helminths were collected as described by Boomker *et al*. (1989*a*, 1989*b*). Briefly, the carcass was opened and the abdominal cavity macroscopically scanned for parasites. Subsequently, the heart, lungs, liver and the oesophagus were examined separately. The remainder of the gastrointestinal tract was divided into rumen and reticulum, abomasum, small intestine and large intestine. Aliquots were taken from the ingesta of each of these sections and the mucosae of the abomasum, small and large intestine digested in pepsin and HCL. The liver and right lung were cut into slices and incubated overnight in normal saline. Digests and incubated samples were washed on 150 *μ*m sieves and sieve residues collected and kept for helminth recovery. Presence or absence of *Taenia* spp. metacestodes was determined by examining incisions of the masseter, triceps and iliopsoas muscles.

Helminths were identified and counted under a compound microscope equipped with differential interference contrast, based on their original descriptions or, if applicable, the latest taxonomic revisions. Where applicable, aliquot counts were converted into total counts. Concerning cestodes, only specimens with the scolex still attached were counted. Members of the Paramphistominae reported from common reedbuck in Africa belong to the genera *Calicophoron* (4 species) and *Cotylophoron* (1 species) (Pfukenyi and Mukaratirwa, 2018). For the present study, specimens were identified to subfamily level. Females of the genera *Cooperia* and *Cooperioides* (Strongylida) are difficult to distinguish morphologically. Consequently, only males were identified to species level, whereas females and fourth-stage larvae were grouped as *Cooperia*-like females and *Cooperia*-like L4, respectively.

Ticks were collected and their burdens determined as set out in Horak *et al*. (1982) and Horak *et al*. (1983), with the exception that only half of each animal was processed for ectoparasite recovery (Horak *et al*., 1988). Briefly, the hides of reedbuck were cut in half and all adult ticks collected, identified and counted and then multiplied by 2. Subsequently, the hides were washed and scrubbed. The water in which the hides had been scrubbed was sieved and subsamples of the collected sievings were examined for immature ticks. Counts were subsequently converted to full counts. Ticks were identified based on their original descriptions or, if applicable, the latest taxonomic revisions.

### Data analysis

As mentioned above, we intentionally analysed the current data in approximately the same way as in Junker *et al*. (2022 for nyalas) to make the results comparable with those of this earlier study. First, we tested whether counts and species richness of helminths and ectoparasites harboured by reedbuck in the 2 localities depended on sex and/or age of a host and differed between localities from which a host was taken. For this part of the analyses, we selected helminth and ectoparasite species that were the most prevalent in both localities, namely (a) the helminths *Cooperia yoshidai*, *Dictyocaulus viviparus*, *Haemonchus contortus*, *Longistrongylus schrenki* and *Setaria bicoronata*; (b) the ticks *Rhipicephalus decoloratus* and *Rhipicephalus evertsi* and (c) a chewing louse, *Damalinia reduncae* and a sucking louse, *Linognathus fahrenholzi* (Supplementary Tables S1 and S2). Data on counts (separately for each of the most prevalent parasite species) as well as on species richness of all parasites (separately for helminths and ectoparasites) were analysed using generalized linear mixed models (GLMMs) with year and season of sampling as random factors (season nested within year). To run the models, we searched for the distribution that best-fitted the data using the package ‘fitdistrplus’ (Delignette-Muller and Dutang, 2015) implemented in R statistical environment (R Core Team, 2022) by fitting negative binomial, geometric and Poisson distributions (all discrete) and then selecting the best distribution based on the Akaike information criterion (AIC) using maximum likelihood estimation as a fitting method. Negative binomial distribution appeared to be the best-fitted for count data, whereas Poisson distribution appeared to be the best-fitted for species richness. Consequently, we ran GLMMs using the R package ‘glmmTMB’ (Brooks *et al*., 2017) with options ‘family = nbinom1’ for counts and ‘family = genpois’ for species richness. For each parasite species or species richness, we fitted the models that included (a) all main effects and all interactions and (b) all possible combinations of independent variables and interactions between them. Then, we selected the best models based on AIC corrected for small sample size (ΔAICc), using function ‘model.sel’ of the R package ‘MuMin’ (Barton, 2020). Among them, we selected models with ΔAICc < 2 and averaged these models using function ‘model.avg’.

Subsequently, we investigated differences in helminth or ectoparasite community composition between female and male adult and young reedbuck from different localities using non-parametric permutational multivariate analysis of variance using distance matrices (PERMANOVA; McArdle and Anderson, 2001) as implemented in function ‘adonis2’ of the R package vegan (Oksanen *et al*., 2020) with the Bray–Curtis dissimilarity and 1000 permutations. To visualize differences in community composition of helminths and ectoparasites in dependence of sex and age of the reedbuck and the locality that they were taken from, we used non-metric multidimensional scaling (NMDS), a non-constrained ordination method (Minchin, 1987) with the function ‘metaMDS’ from the ‘vegan’ package. In contrast to other ordination methods in which many axes are calculated, but only a few are viewed due to graphical limitations, in NMDS a small number of axes are explicitly chosen prior to the analysis and the data are fitted to those dimensions, so there are no hidden axes of variation (Borcard *et al*., 2018). For this, we constructed 2 presence/absence matrices with helminth or ectoparasite species as columns and reedbuck individuals as rows. This was done because (a) counts cannot be compared between parasites belonging to different species, families and phyla and (b) parasite incidence data have been shown to represent information no less reliably than parasite count data for investigations of parasite community ecology, at least at the scale of parasite infracommunities (Krasnov *et al*., 2021).

To explore patterns of compositional and functional similarity in helminth or ectoparasite species composition among female and male adult and young reedbuck from different localities, we used double similarity principal component analysis (DSPCA), a recently developed ordination method (Pavoine, 2019). In brief, DSPCA places species (in our case, parasites) and communities (in our case, infracommunities of hosts belonging to a given sex/age cohort from a given locality) in the same ordination space and identifies those parasites that drive similarity patterns in accordance with their occurrence or traits. First, DSPCA takes a matrix of compositional or trait-based similarity between parasite species, eigen-decomposes this matrix, produces a series of independent axes and places species into the space of these axes according to their compositional or trait-based similarity. Then, the matrix of parasites by host cohorts is used for placement of host cohorts in the centres of the parasites that they harbour and distributing them in this space so that the host cohorts are positioned in the ordination space according to the coordinates of the parasites they harboured. Following this, DSPCA produces new axes (principal components) that best describe the similarities between host cohorts based on composition of their parasite communities, and the coordinates of parasites and host cohorts are projected on these principal components which reflect (from the first to the last) the ever-decreasing portion of similarities and, concomitantly, the ever-increasing portion of dissimilarity in parasite species/trait composition between host cohorts. In the final ordination space of DSPCA, the coordinates of host cohorts and parasites are bounded between −1 and 1 with parasites and host cohorts being displayed by arrows, starting from the origin. A more acute angle between the arrows of any 2 host cohorts reflects higher similarity between them in terms of parasite species composition or parasite traits. An arrow of a host cohort is directed to the centre of its parasite species. An arrow of a parasite is directed towards the host cohorts in which it occurs, whereas its length reflects how well this parasite represents the composition of each parasite community in a host cohort.

Given that traits of helminths and ectoparasites are vastly different, we (a) constructed separate community matrices for helminths and ticks (see below), with reedbuck of different sex/age cohorts and taken from different localities in rows and parasite species in columns, with prevalence of a given parasite in a given cohort from a given locality as entries and (b) parasite trait matrices with parasite species as rows and traits as columns separately for each trait. For helminths, these traits (see Supplementary Table S3) were (a) life cycle (direct or indirect; 2 binary variables); (b) preferred site of parasitism within the host (9 binary variables) and (c) transmission mode (trophic, vector-borne, actively invading; 3 binary variables). For ticks, the traits (see Supplementary Table S4) were (a) preferred site of attachment of an adult tick (caudal region, underside, ears or any part of the body; 4 binary variables); (b) difference between main host of an adult and an immature stage (different or the same; 2 binary variables); and (c) seasonality of adults (summer, spring-summer, spring, winter or all year round; 5 binary variables). We did not apply DCPCA for lice because there were only 2 louse species which differed in a single main trait (feeding on either blood or epidermis of a host). Then, we calculated the compositional or functional (for each trait separately) similarity between parasite species found in reedbuck of different sex/age cohorts from different localities using the SOchiai index of similarity developed by Pavoine and Ricotta (2014) and implemented in the functions ‘dsimTree’ (for compositional similarity) and ‘dsimFun’ (for functional similarity) of the R package ‘adiv’ (Pavoine, 2020). Subsequently, we ran the DCPCA for species composition or each trait using the function ‘dspca’ of the package ‘adiv’.

At the final stage of the analyses, we considered individual-based reedbuck–helminth and reedbuck–ectoparasite networks and (a) estimated the non-random structural patterns in these networks by calculating their nestedness (in our case, a pattern in which hosts exploited by more specialized parasites form a proper subset of those taken by more generalist parasites; Bascompte *et al*., 2003); (b) assessed importance of individual reedbuck in each network using 3 indices, namely index of individual host specialization (*d’*), individual host strength (IHS) and centrality (C) (see explanations below); and (c) tested for the associations between sex and age of a reedbuck individual and a locality from which it was taken and each of these indices. For each locality and separately for helminth and ectoparasites, we constructed 5 interaction binary matrices with individual hosts in rows and parasites in columns, namely a matrix with all reedbuck, a matrix with female reedbuck only, a matrix with male reedbuck only, a matrix with adult reedbuck only and a matrix with young reedbuck only.

Nestedness of each network was calculated based on the NODF index (Nestedness based on Overlap and Decreasing Fill; Almeida-Neto *et al*., 2008), using function ‘nestednodf’ implemented in the R package ‘vegan’. We then tested for significance using function ‘oecosimu’ of ‘vegan’ with null model ‘r1’ (preserves row frequencies and uses column marginal frequencies as probabilities of selecting species) and 1000 permutations. NODF varies from 0 (random network) to 100 (perfect nestedness). The indices of host importance in the networks were as follows. Individual host specialization (*d’*) compares the frequency distribution of host–parasite interactions with a null distribution when interactions between hosts and parasites are proportional to their observed total frequencies and may vary from 0 (no specialization) to 1 (complete specialization) (Blüthgen *et al*., 2006; Blüthgen, 2010). In other words, it describes the deviation from a neutral configuration of associations. IHS is the species-strength index of Bascompte *et al*. (2006) calculated as the sum of the dependencies of each parasite on each individual host. *d’* and IHS were calculated using function ‘specieslevel’ of the R package ‘bipartite’ (Dormann *et al*., 2008; Dormann, 2011). The centrality metric estimates the role of each host in sharing parasites with other hosts. A higher centrality of a host reflects its greater connection with other hosts and, thus, high level of parasite transmission in the network (Morand *et al*., 2014; Pilosof *et al*., 2015). To calculate centrality, we projected each bipartite network to a unipartite network using the function ‘projecting_tm’ of the R package ‘tnet’ (Opsahl, 2009), transformed the latter into the network graph object using function ‘tnet_igraph’ of the ‘tnet’ and then calculated centrality as eigenvector centrality using function ‘evcent’ of the R package ‘igraph’ (Csardi and Nepusz, 2006). Associations between sex and age of a reedbuck and a locality from which it was taken and each of the above 3 indices were tested using generalized linear models (GLMs) with these factors as explanatory variables and each index as a response variable. GLMs for *d’* and centrality were run using the option ‘family = quasibinomial’ because these indices vary between 0 and 1.

## Results

### Helminth communities: general overview

Helminths of infracommunities of common reedbuck in KwaZulu-Natal Province belonged to 18 species of nematodes, 1 subfamily of trematodes (Paramphistominae) and 2 species of cestodes (Supplementary Table S1). Of these, 13 species were present at HM, and reedbuck there had a mean species richness of 4.4, ranging from 0 to 8, with a single young female harbouring no helminths. Eighteen helminth species were collected at ESNR and mean species richness was 5.3, ranging from 1 to 12. Seven species of nematodes, Paramphistominae and the cestode *Moniezia benedeni* were shared between the 2 sites, whereas *Ostertagia ostertagi*, *Trichostrongylus falculatus*, *Setaria labiatopapillosa* and metacestodes of *Taenia hydatigena* were only found at HM, where they had a prevalence of 15% or less. In contrast, 8 species, *Cooperia hungi*, *Cooperioides hepaticae*, *Cooperia*-like females, *Gongylonema* sp., *Impalaia tuberculata*, *Oesophagostomum columbianum*, *Setaria* sp. and *Skrjabinema* sp., only infected reedbuck at ESNR. Of these, *Setaria* sp. and *Skrjabinema* sp. had a prevalence of 20 and 37%, respectively, while the prevalence of the remaining species was below 15% (Supplementary Table S1). Five species of nematodes were common and abundant members of the helminth communities of reedbuck at both localities and had a prevalence exceeding 50% (42% for *H. contortus* at HM). These were *C. yoshidai*, the most prevalent helminth (89%) at HM, the abomasal nematodes *H. contortus* and *L. schrenki*, the most prevalent helminths (93%) at ESNR, as well as the lung worm *D. viviparus* and the filarial nematode *S. bicoronata*. In addition, Paramhistominae were abundant and had a high prevalence (65%) at HM, but only infected a single animal at ESNR.

### Ectoparasite communities: general overview

Ectoparasite communities in KwaZulu-Natal Province comprised 11 species of ixodid ticks, 1 species of chewing lice, *D. reduncae*, and 1 species of sucking lice, *L. fahrenholzi* (Supplementary Table S2). Both louse species occurred at HM and ESNR, but prevalence as well as abundance of both adult and immature stages of these parasites were higher at the former locality. In addition, prevalence of adults was higher than that of immature stages in both louse species, irrespective of locality. A total of 7 ectoparasite species were found on reedbuck at HM, and mean species richness was 3.2, with a range of 1–6, whereas 11 species of ectoparasites were collected at ESNR, with a mean species richness of 3.5 and a range of 1–8. Adults of 3 species of ticks, *Ixodes* sp., *R. evertsi evertsi* and *R. lounsburyi,* infested reedbuck at HM, but prevalence for all 3 species was below 10%. Immatures of *Ixodes* sp. and *R. evertsi evertsi* at HM were more prevalent (36 and 88%, respectively), but no immatures of *R. lounsburyi* were found. However, immatures of *R. appendiculatus* and *R. decoloratus* had a prevalence of 8 and 36%, respectively. The only adult tick species shared between HM and ESNR was *R. evertsi evertsi*. At ESNR, the adults of *R. appendiculatus*, *R. decoloratus* and *R. muehlensi* were recovered, with a prevalence ranging from 8 to 19%. The immatures of 9 tick species were present at ESNR with only those of *Ixodes* sp. and *R*. *lounsburyi* being absent from this locality. As at HM, the immature stages of a particular tick species were more prevalent than adults. Irrespective of the developmental stage, 2 tick species, *Ixodes* sp. and *R. lounsburyi*, were only found at HM, whereas 6 tick species were exclusively collected from reedbuck at ESNR, namely *Amblyomma hebraeum*, *Amblyomma marmoreum*, *Haemaphysalis* sp., *Rhipicephalus maculatus*, *R. muehlensi* and *Rhipicephalus* sp. Shared species between the 2 sites were *R. appendiculatus*, *R. decoloratus* and *R. evertsi evertsi*.

### Effect of host sex/age and locality on parasite abundance

**Figure 1.**
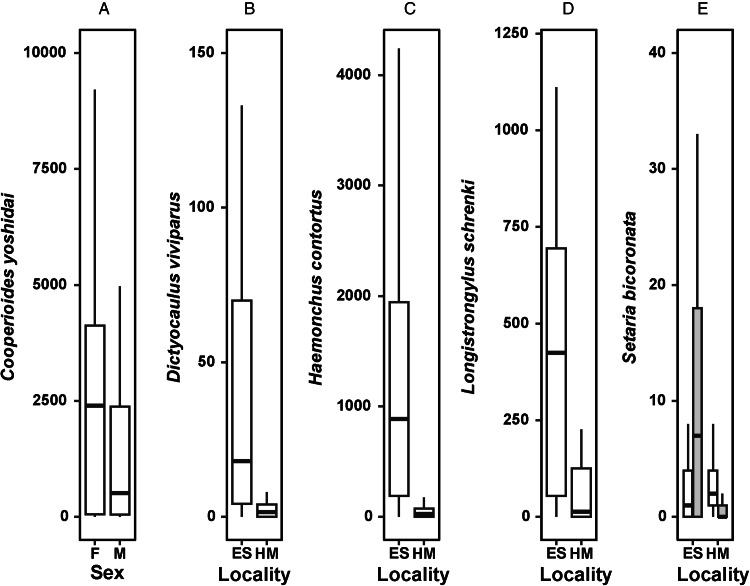
Median (horizontal line), lower and upper quartiles (boxes) and maximal and minimal numbers (whiskers) of (A) *Cooperia yoshidai* in female and male reedbuck; (B, C, D) *Dictyocaulus viviparus*, *Haemonchus contortus* and *Longistrongylus schrenki*, respectively, in reedbuck from the Eastern Shores Nature Reserve (ES) and Himeville (HM); and (E) *Setaria bicoronata* in adult (white boxes) and young (grey boxes) reedbuck from the Eastern Shores Nature Reserve (ES) and Himeville (HM).

The counts of 3 of 5 of the most prevalent helminths differed significantly between localities only, with *D. viviparus*, *H. contortus* and *L. schrenki* being more numerous in reedbuck from ESNR than from HM (Table 1, Fig. 1). Female reedbuck harboured higher numbers of *C. yoshidai* than their male counterparts (Table 1, Fig. 1). The counts of *S. bicoronata* were affected by the interaction between a reedbuck's age and a locality but not by either factor separately (Table 1). In particular, significantly more individuals of *S. bicoronata* were collected from young reedbuck in ESNR than in Himesville, whereas no locality difference in the counts of this nematode was found in adult reedbuck (Fig. 1).

**Table 1.** Averaged coefficients of the best generalized linear mixed-effects models with the negative binomial distribution of the effect of a reedbuck's sex (SX), age (A) and locality (Loc) from which a reedbuck was taken and their interactions on the numbers of the most prevalent helminth (H) and ectoparasite (EC) taxa

H/EC		Explanatory variable	Coefficient ± S.E	*z*	*P*
H	*Cooperia yoshidai*	SX (males)	−0.65 ± 0.32	−2.03	0.04
	*Dictyocaulus viviparus*	Loc (Himeville)	−1.41 ± 0.35	−4.07	0.004
	*Haemonchus contortus*	Loc (Himeville)	−2.10 ± 0.36	−5.81	<0.001
	*Longistrongylus schrenki*	Loc (Himeville)	−1.51 ± 0.32	−4.74	<0.001
	*Setaria bicoronata*	A (young) × Loc (Himeville)	−2.48 ± 0.06	−2.61	0.01
EC	*Rhipicephalus decoloratus*	A (young)	−0.59 ± 0.34	−1.71	0.08
	*Damalinia reduncae*	SX (males)	1.43 ± 0.80	1.79	0.07
		Loc (Himeville)	1.89 ± 0.78	2.43	0.01
	*Linognathus fahrenholzi*	Loc (Himeville)	1.05 ± 0.49	2.16	0.03

Random factor in all models was year and season of sampling. Reference levels of independent variables were female for sex, adult for age and the Eastern Shores Nature Reserve for locality. Only significant and marginally significant coefficients are shown.

**Figure 2.**
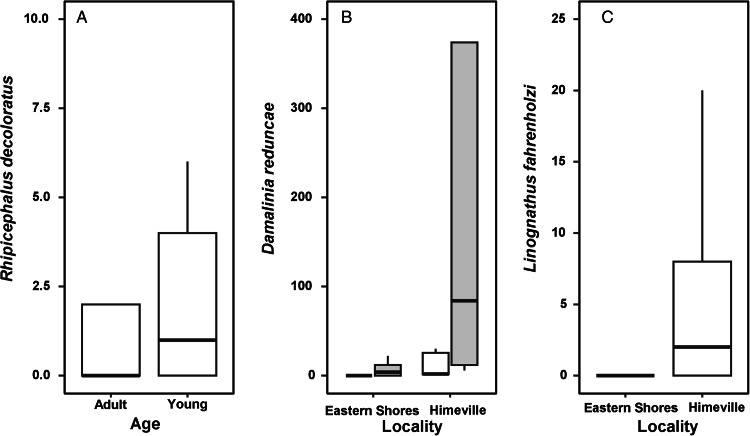
Median (horizontal line), lower and upper quartiles (boxes) and maximal and minimal numbers (whiskers) of (A) *Rhipicephalus decoloratus* in adult and young reedbuck; (B) *Damalinia reduncae* in female (white boxes) and male (grey boxes) reedbuck from the Eastern Shores Nature Reserve (ES) and Himeville (HM); and (C) *Linognathus fahrenholzi* in reedbuck from the Eastern Shores Nature Reserve and Himeville.

For the most prevalent ticks, we found a marginally significant difference in the numbers of *R. decoloratus* only, with a weak trend of counts being higher in young than adult reedbuck (Table 1, Fig. 2). Counts of the chewing louse *D. reduncae* differed significantly between the reedbuck from the 2 localities, being higher in HM than in ESNR (Table 1, Fig. 2). In addition, a marginally significant effect of a reedbuck's sex on the number of these lice reflected a tendency of males harbouring higher numbers of *D. reduncae* than females (Table 1, Fig. 2). Counts of the sucking louse *L. fahrenholzi* differed significantly between localities only, being higher in reedbuck from Himesville than from ESNR (Table 1, Fig. 2).

### Effect of host sex/age and locality on parasite species richness and species composition

A single model returned for helminth species richness suggested no effect of either host sex or locality and only a marginally significant effect of host age (coefficient = −0.21 ± 0.12, *z* = 1.71, *P* = 0.08), with the number of helminth species tending to be higher in adult animals (note reference level and the sign of the coefficient). In contrast, adult and young female and male reedbuck harboured similar numbers of ectoparasite species in both localities.

**Figure 3.**
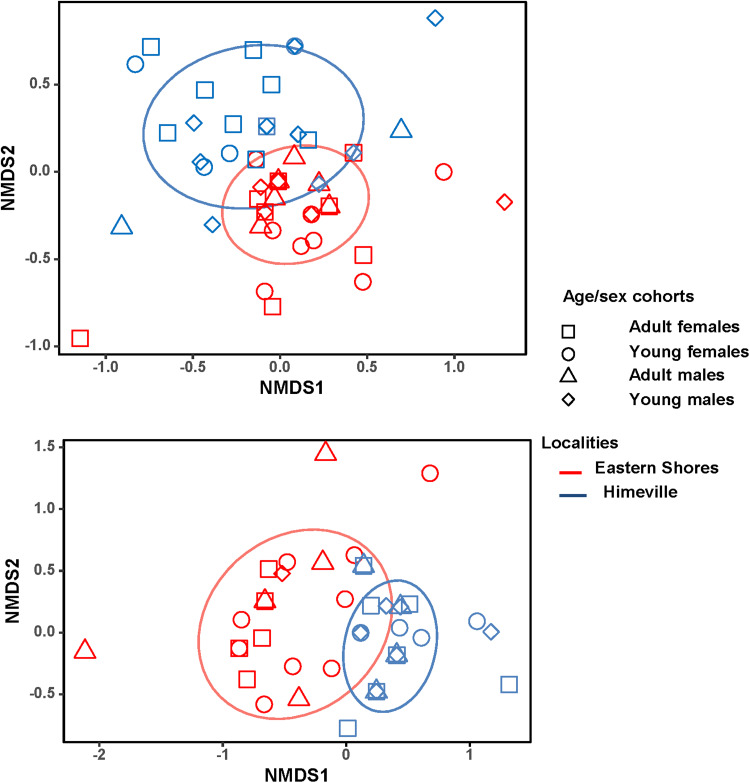
Biplots of non-metric multidimensional scaling of species composition of helminth (A) and ectoparasite (B) infracommunities harboured by adult and young female and male reedbuck in the Eastern Shores Nature Reserve and Himeville.

The results of PERMANOVA are presented in Table 2 and visualization of these results using NMDS is presented in Fig. 3. Helminth species composition in the infracommunities harboured by reedbuck differed solely between localities. Similarly, the effect of locality on differences in ectoparasite species composition was the strongest, although there was also a weaker effect of reedbuck age and a marginal effect of host sex (Fig. 3). In other words, both helminth and ectoparasite species composition differed mainly between localities.

**Table 2.** Results of PERMANOVA of species composition (counts of each species) in helminth (H) and ectoparasite (EC) communities harboured by adult and young male and female reedbuck from 2 localities

H/EC	Factor	d.f.	Sum of squares	*R*^2^	*F*	*P*
H	Age	1	0.19	0.03	2.12	0.09
	Sex	1	0.00	0.00	−0.02	0.96
	Locality	1	1.05	0.18	11.66	<0.001
	Age × sex	1	0.07	0.01	0.80	0.54
	Age × locality	1	0.16	0.03	1.73	0.15
	Sex × locality	1	−0.01	0.00	−0.13	0.97
	Age × sex × locality	1	0.20	0.03	2.21	0.09
	Residual	47	4.23	0.72		
	Total	54	5.88	1		
EC	Age	1	0.37	0.04	2.91	0.04
	Sex	1	0.31	0.04	2.42	0.07
	Locality	1	1.78	0.21	13.99	<0.001
	Age × sex	1	0.12	0.01	0.91	0.46
	Age × locality	1	0.22	0.03	1.71	0.18
	Sex × locality	1	0.04	0.00	0.32	0.81
	Age × sex × locality	1	0.09	0.01	0.73	0.58
	Residual	43	5.46	0.65		
	Total	50	8.38	1

### Effect of host and parasite traits on parasite infracommunities

**Figure 4.**
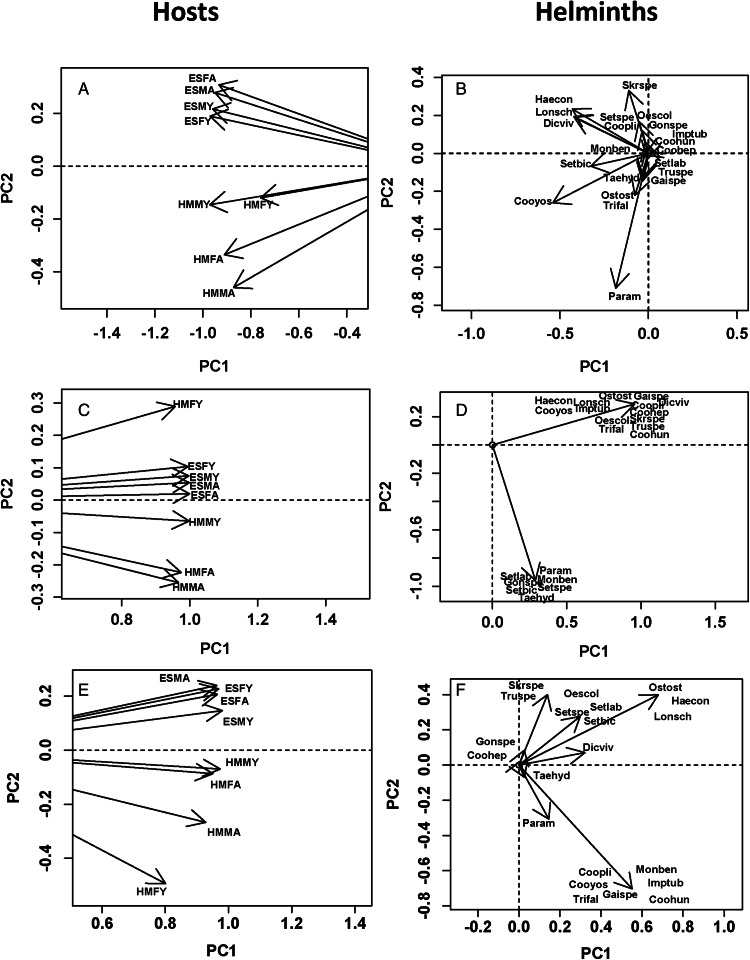
Results of DSPCA applied to helminth infracommunities in adult (ad in community label) and young (y in community label) male (M in community label) and female (F in community label) reedbuck from the Eastern Shore Nature Reserve (ES in community label) and Himeville (HM in community label), based on parasite prevalence in sex/age cohorts from the 2 localities either considering helminth species as maximally dissimilar (A, B) or based on similarities in a helminth's life cycle (C, D) or based on similarities among helminths according to preferred site within a host (E, F) (see text for details). Hosts: host scores on principal components PC1 and PC2; helminths: parasites scores on principal components PC1 and PC2. See Supplementary Table S1 for parasite species names.

**Figure 5.**
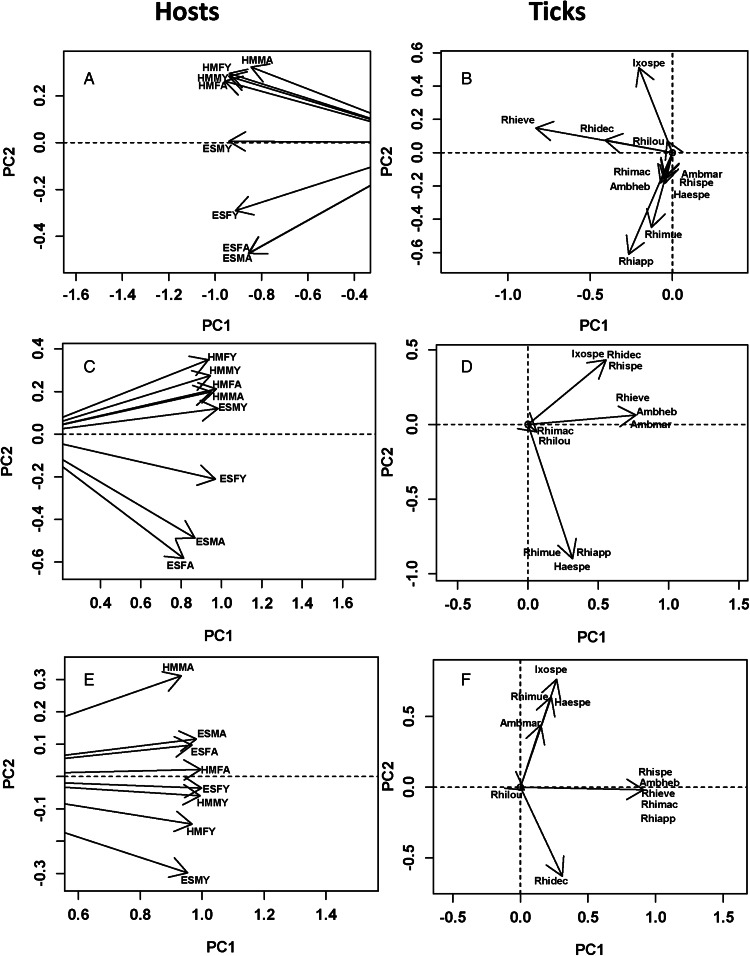
Results of DSPCA applied to tick communities in adult (ad in community label) and young (y in community label) male (M in community label) and female (F in community label) reedbuck from the Eastern Shore Nature Reserve (ES in community label) and Himeville (HM in community label), based on parasite prevalence in sex/age cohorts from the 2 localities either considering tick species as maximally dissimilar (A, B) or based on similarities among tick species according to their preferred sites of attachment (C,D) or based on similarities in seasonal patterns of occurrence (E, F) (see text for details). Hosts: host scores on principal components PC1 and PC2; Ticks: parasites scores on principal components PC1 and PC2. See Supplementary Table S2 for parasite species names.

Helminth and tick infracommunities were similar between adult and young male and female reedbuck from the 2 localities in terms of species composition (0.80 and 0.81, respectively). Trait-based similarities between infracommunities were even higher (Table 3). DSPCA demonstrated high similarities among host cohorts within each locality. Given that each DSPCA produced only 1 eigenvalue >1 (Table 3), helminth and tick infracommunities of all sex/age cohorts from both localities had similar scores on the PC1 for all DSPCA (Figs 4–5), but the distribution of infracommunities along PC2 demonstrated a certain degree of dissimilarity. For example, when DSPCA was based solely on species composition, helminth and tick infracommunities of reedbuck from ESNR and those from HM had PC2 scores of the opposite signs (Figs 4A and 5A). The distribution of helminth infracommunities based on life cycle also showed differences between infracommunities of reedbuck from ESNR and HM (positive and negative, respectively, scores on PC2; Fig. 4C). However, infracommunities of young females in HM differed from those of the remaining sex/age cohorts there and were similar to those of the animals from ESNR. The ordination diagram of helminths (Fig. 4D) suggested that infracommunities from ESNR were dominated by helminths with direct life cycles, whereas those from HM were characterized by the comparatively greater presence of species requiring intermediate hosts (Supplementary Table S3). The distribution of helminth infracommunities based on predilection site again showed differences between infracommunities of the animals from ESNR and HM (Fig. 4E), with the former harbouring several species of parasites occurring in the large intestine, whereas the latter infracommunities were characterized by the high prevalence of helminths residing in the small intestine and rumen (Paramphistominae) (Fig. 4F).

**Table 3.** Mean similarity (MS) in helminth (H) and tick (TK) communities of reedbuck and eigenvalues produced by double similarity principal component analysis (DSPCA) for trait similarity in these communities (see text for details)

H/TK	Trait	MS	*N*_t_	*N*_1_	1^st^ eigenvalue	Last eigenvalue
H	Life cycle strategy	0.97	8	1	7.78	0.01
	Preferred site in host	0.93	8	1	7.48	0.001
	Transmission mode	0.97	3	1	7.79	0.03
TK	Preferred site in host	0.84	4	1	6.94	0.02
	Seasonality	0.94	5	1	7.59	0.001
	Host shift between adults and immatures	0.97	2	1	7.79	0.21

*N*_t_, total number of eigenvalues; *N*_1_, number of eigenvalues >1

When similarity among ticks in terms of the preferred attachment site was considered, ticks preferring to attach to the ear pinnae of a host were mainly characteristic for reedbuck of ESNR except for young males (Supplementary Table S4). The latter as well as reedbuck from HM were mainly attacked by ticks preferring either the caudal region of the body or the underside or without clear preference (Fig. 5C and D). Regarding between-tick similarity in seasonal patterns of occurrence, DSPCA demonstrated that young animals at either locality were predominantly parasitized by *R. decoloratus* mostly occurring in spring (negative scores on PC2), whereas adult reedbuck harboured tick species occurring in summer, spring-summer, winter or all year round (Fig. 5E and F).

### Reedbuck–parasite networks

**Figure 6.**
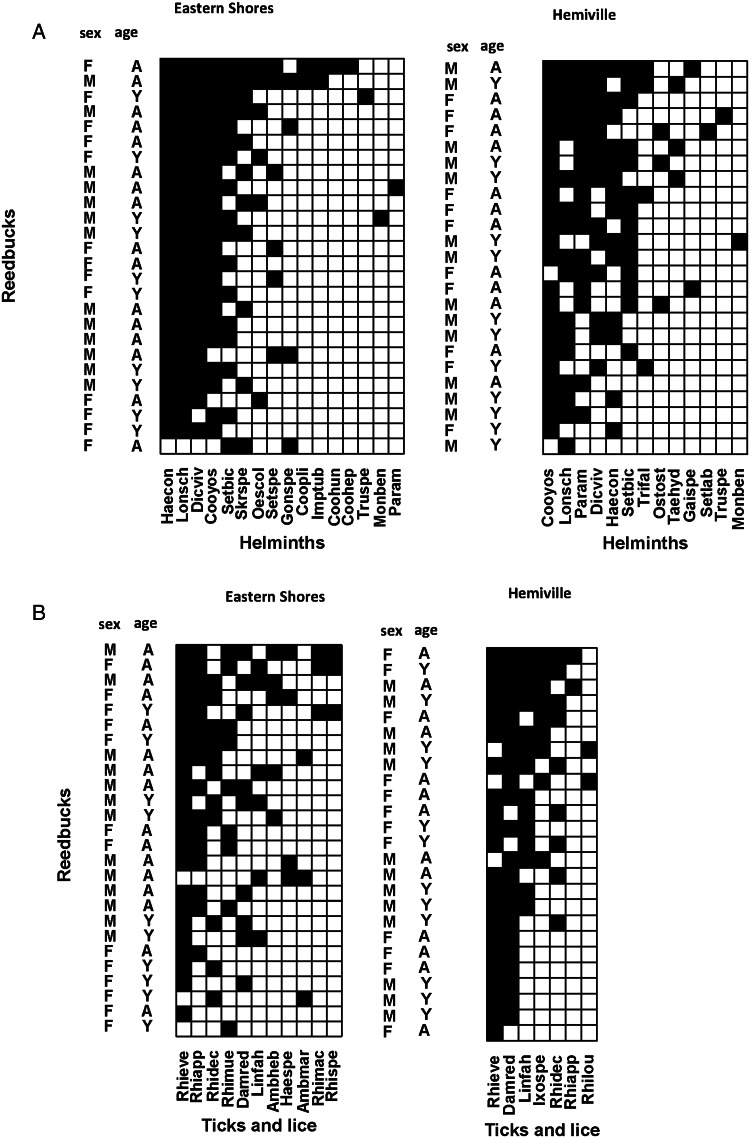
Nested (i.e. sorted by row/column sums) network matrices of (A) individual reedbuck–helminth and (B) reedbuck–ectoparasite associations in 2 localities based on presence/absence data. M and F, male and female hosts, respectively; A and Y, adult and young hosts, respectively. See Supplementary Tables S1 and S2 for parasite species names.

**Figure 7.**
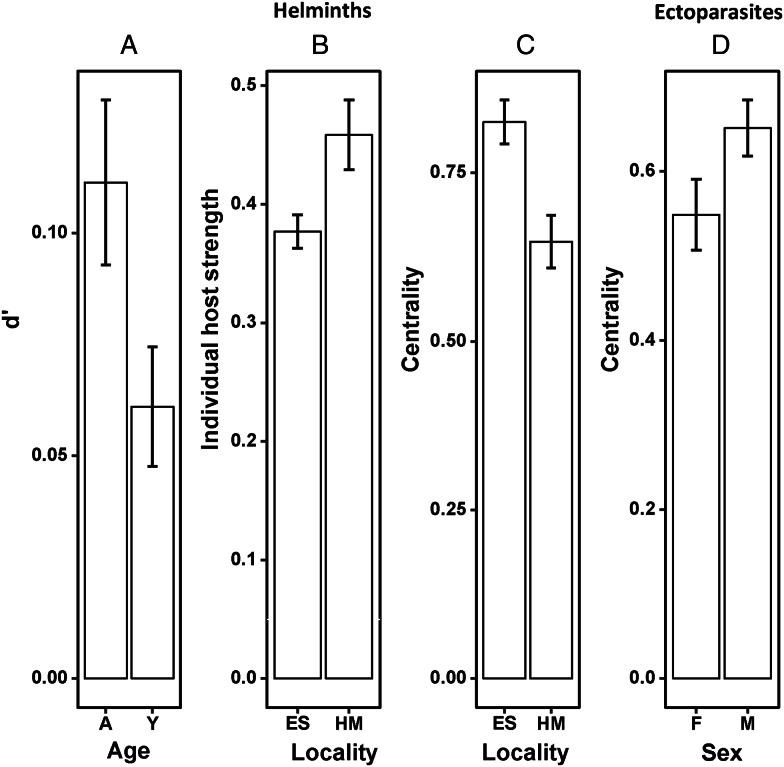
Mean (±s.e.) values of (A) individual host specialization (*d’*) of adult and young reedbuck in reedbuck–helminth networks; (B, C) individual host strength and centrality, respectively, of reedbuck from the Eastern Shores Nature Reserve (ES) and Himeville (HM); (D) centrality of female (F) and male (M) reedbuck in reedbuck–ectoparasite networks.

Individual-based reedbuck–parasite networks in the 2 localities are visualized in Fig. 6. In the ESNR, reedbuck–helminth networks were significantly nested for male and adult antelopes only, whereas in HM these networks were significantly nested when all animals were considered as well as for separate male and female networks (Table 4). In contrast, reedbuck–ectoparasite networks in both localities were significantly nested except female and young reedbuck in HM (Table 4). Summaries of GLM for the effect of a reedbuck's sex, age and locality on the values of network indices are presented in Table 5. In reedbuck–helminth networks, values of individual host specialization (*d’*) differed and were significantly higher in adult than young hosts (Fig. 7A), values of IHS and centrality differed between localities with the former being higher in HM than in the ESNR, whereas the opposite was true for the latter (Fig. 7B and C). Finally, in reedbuck–ectoparasite networks, the effect of a host's sex, age or locality was found for centrality only, with males being more central than females (Fig. 7D).

**Table 4.** Nestedness (NODF; see text for explanation) of individual-based reedbuck-helminth (H) and reedbuck–ectoparasite (EC) networks that include either all reedbuck individuals or males only, females only, adults only or young animals only from the 2 localities, the Eastern Shores Nature Reserve. and Himeville

H/EC	Locality	Reedbuck in the network	NODF	*P*
H	Eastern Shores	All	48.88	0.05
		Males	41.35	0.03
		Females	44.44	0.23
		Adult	50.97	0.01
		Young	30.94	0.55
	Himeville	All	56.37	0.01
		Males	44.03	0.01
		Females	41.75	0.03
		Adult	42.41	0.05
		Young	38.78	0.07
EC	Eastern Shores	All	54.73	0.01
		Males	36.01	0.01
		Females	40.2	0.01
		Adult	44.06	0.01
		Young	25.29	0.01
	Himeville	All	43.76	0.03
		Males	33.02	0.03
		Females	22.61	0.13
		Adult	37.73	0.03
		Young	25.62	0.07

NODF varies from 0 (random network) to 100 (perfect nestedness). Significance of each NODF value was determined using null model with 1000 permutations.

**Table 5.** Summary of generalized linear models of the effect of a reedbuck's sex (SX), age (A) and locality (Loc) on individual reedbuck specialization (*d’*), individual host strength (IHS) and centrality (C) in individual-based reedbuck endo- (EN) and ectoparasites (EC) from 2 localities (see text for explanations)

EN/EC	Dependent variable	Explanatory variable	Coefficient ± s.e.	*t*	*P*
EN	*d’*	A (young)	−0.05 ± 0.02	−1.99	0.05
	IHS	Loc (Himeville)	0.08 ± 0.03	2.41	0.02
	C	Loc (Himeville)	−0.18 ± 0.05	−3.49	<0.01
EC	C	SX (males)	0.10 ± 0.05	1.91	0.06

Models for *d’* and C were with quasibinomial distribution and logit link. Models for IHS were with Gaussian distribution and log link. Reference levels of independent variables were female for sex, adult for age and the Eastern Shores Nature Reserve for locality. Only significant and marginally significant coefficients are shown.

## Discussion

Comprising a total of 21 helminth and 13 ectoparasite species, the parasite fauna of reedbuck in KwaZulu-Natal was moderately species rich. Despite our expectation that higher gregariousness in nyalas would lead to higher species richness when compared to predominantly solitary reedbuck, overall species richness did not differ much in the helminth communities of the 2 hosts. Helminth communities of nyalas comprised a total of 19 species, with an overall species richness at the 3 localities examined of 3.5, ranging from 0 to 7 (Junker *et al*., 2022). Both helminth communities were dominated by directly transmitted nematodes. The high density of reedbuck, especially at ESNR (Horak *et al*., 1988), might have resulted in increased contact rates between reedbuck individuals.

As predicted, and likely linked to the low sociality in all cohorts studied, host age and sex affected host–parasite interactions in reedbuck less than in nyalas. Environmental conditions, however, had a strong influence on helminth community structure in both these hosts. Furthermore, similar to helminth communities in nyalas, host age was more important in driving community and network structure than host sex, and different parasite species were affected differently by host sex, age or the locality in which they were collected (Junker *et al*., 2022).

Both the helminth and ectoparasite faunas of reedbuck were more diverse at ESNR than at HM. While 9 species of helminths were shared between reedbuck at HM and ESNR, their species complement varied not only in prevalence and abundance, but also in the presence/absence of certain species at a given locality. *Cooperiodes hepaticae*, for example, was present only at ESNR. This liver parasite is predominantly a parasite of impala (Boomker *et al*., 1989*b*), which shared the habitat of reedbuck at ESNR but not at HM, emphasizing the influence of cross-infection and parasite sharing between suitable host species and the role of reservoir hosts in parasite transmission (Boomker *et al*., 1989*b*; Fenton *et al*., 2015; Dallas *et al*., 2019; Horak *et al*., 2021). Helminth species shared between the 2 localities generally had lower prevalence and abundance at HM than ESNR. The harsher climatic conditions at HM, including colder and drier winters, could have impeded the survival of free-living stages of gastrointestinal Strongylida that have minimum humidity and temperature thresholds for their development (Rossanigo and Gruner, 1995; O'Connor *et al*., 2006). Similarly, the wider spectrum of co-occurring hosts and different habitat conditions may have led to the larger variety seen in ixodid tick species at ESNR in relation to HM. Furthermore, livestock at Himeville were dipped on a regular basis to control tick infestations (Horak *et al*., 1988), decreasing the likelihood of domestic animals serving as maintenance hosts for ticks.

As for nyalas (Junker *et al*., 2022), the effects of sex, age and locality on parasite numbers differed between parasite species. A significant effect of host sex on helminth abundance was only seen in *C. yoshidai*, with females supporting larger numbers than males. Variation of helminth prevalence and intensity between hosts of different age and sex classes has been attributed to differential exposure and transmission rates, brought about by different behavioural patterns, habitat use, body size and/or immune competence (Amundson *et al*., 2016). Female bias in parasitism can be associated with higher parental care cost (Sitko and Heneberg, 2015). Female reedbuck may consume more forage to meet the heightened energy demands of parturition and suckling, increasing exposure rates to trophically transmitted parasite stages. In addition, suckling females are confined to a narrow habitat range while their lambs are hidden for a period of about 3 months (Skinner and Chimimba, 2005), which may increase levels of contamination in the vicinity.

The interaction between host age and locality resulted in higher numbers of *S. bicoronata* in young reedbuck at ESNR when compared to HM. *Setaria* species are vector-born parasites, transmitted by haematophagous insects, mostly mosquitoes (Cancrini *et al*., 1997; Anderson, 2000), whose populations can be limited by temperature (Guarido *et al*., 2021). Higher vector abundance at the ESNR, combined with lower immune competence in young reedbuck, may be responsible for the observed pattern.

With regard to ectoparasites, there was a weak but significant effect of host age on the abundance of *R. decoloratus*, reaching higher numbers in young animals. Cattle may have innate and acquired immunological responses to infestation with *R. decoloratus* and difference in coat characteristics is considered an important contributor to variation in resistance against this tick in various cattle breeds (Marufu *et al*., 2011; Marima *et al*., 2020). Acquired immunity in adults and differences in coat characteristics, as evidenced by changes in colour patterns between young and adult reedbuck (Skinner and Chimimba, 2005), might result in higher burdens of *R. decoloratus* in young animals.

Host age had a marginally significant effect on helminth species richness, with a larger variety of species found in adult reedbuck, possibly because of higher vagility, increased food intake or a time-dependent increase in exposure to infective stages of parasites in older animals (Chaisiri *et al*., 2017; Bellay *et al*., 2020).

As previously shown for small mammals (Behnke *et al*., 2001, 2004), the main differences concerning parasite communities in reedbuck were found between localities. Similarly, species composition of infracommunities (based on taxonomy as well as parasite traits) differed mostly between localities. It was the only effect regarding helminth infracommunities and the strongest effect with regard to ectoparasite infracommunities.

Considering life cycle characteristics, helminth infracommunities at ESNR mostly encompassed helminths with direct life cycles, whereas helminths with indirect life cycles played a comparatively larger role in infracommunities at HM. The exception to this were young females at HM, whose infracommunities were similar to those of hosts at ESNR. While infracommunities at both localities included 5 indirect life cycle species each, at HM these formed part of a total helminth species complement of 13, compared to a total of 18 species at ESNR. Furthermore, Paramphistominae, which had a prevalence of 65% at HM, infected only 1 host at ESNR. Junker *et al*. (2022) attributed local differences in the prevalence of Paramphistominae in nyalas to variation in the distribution of the intermediate freshwater snail host. Crop irrigation, contributing to snail survival, and high livestock densities have been associated with high infection rates with Paramphistominae (Pfukenyi and Mukaratirwa, 2018). Both conditions were met at HM.

Tick infracommunities on reedbuck at ESNR were characterized by species preferring the ear pinnae of their host as attachment site. This pattern is mainly generated by the higher prevalence of *R. appendiculatus* on reedbuck at ESNR as well as the presence of *R. muehlensi* and *Haemaphysalis* sp., both of which were absent at HM. These tick species mostly attach to the ear pinnae. Both *R. appendiculatus* and *R. muehlensi* are generalists, their adults and immatures infesting large domestic and wild ruminants. However, nyalas, and probably bush buck, both of which were present at ESNR but absent at HM, are preferred hosts of all developmental stages of *R. muehlensi* (Horak *et al*., 1995).

The seasonality of adult ticks affected tick distribution patterns at both localities in a similar way, with infracommunities on young animals being characterized by *R. decoloratus*, a tick of which the highest burdens are usually recorded in spring on wild herbivores in southern Africa (Walker *et al*., 2003; Horak *et al*., 2018). *Rhipicephalus decoloratus* is the only 1-host tick among the tick fauna of reedbuck at the 2 localities. It is possible that the shortened life cycle of 1-host ticks when compared to 2- or 3-host ticks (Spickett, 2013) allows this species to be first to infect young animals.

The structure of reedbuck–helminth and reedbuck–ectoparasite networks also varied mainly between localities, but the 2 networks showed opposite trends. At ESNR, reedbuck–helminth networks were significantly nested for male reedbuck and adults. Higher feeding rates in adults and males could contribute to the nested patterns seen in these host cohorts (González and Poulin, 2005; Bellay *et al*., 2020). During rut, dominant males in the highland regions of KwaZulu-Natal defend territories closest to the food source (Skinner and Chimimba, 2005). These would typically be areas that attract higher host densities and therefore might have higher contamination levels as well as variety of parasite species.

Reedbuck–ectoparasite networks were typically nested, with the exception of those of females and young reedbuck at HM. The reasons behind the seemingly random accumulation of ectoparasites in these 2 host groups at HM are unclear.

Individual host specialization *d’* with respect to helminths was higher in adult than young reedbuck. The opposite was found in nyala and sperm whale populations, where host specialization decreased with host age (Bellay *et al*., 2020; Junker *et al*., 2022). Acquired immunity to helminth infection has been reported in several vertebrate hosts (Tinsley *et al*., 2012), including strongyle parasites in ruminants (Vercruysse and Claerebout, 1997). A naïve immune system combined with habitat shift and raised stress levels when young reedbuck leave their original family group could increase exposure and susceptibility to different parasite species.

Host strength was influenced by locality and was higher in reedbuck at HM when compared to ESNR. Species composition varied significantly between the 2 localities, likely because different parasite species might have differential environmental tolerances as well as final and intermediate host density thresholds (Arneberg, 2002; Calvete *et al*., 2004; O'Connor *et al*., 2006; Cardoso *et al*., 2021).

Centrality was higher in reedbuck at ESNR than at HM. Gómez *et al*. (2013) suggested that a higher probability of encounters between hosts would enhance conditions of parasite sharing. Population densities of reedbuck were especially high at ESNR (Horak *et al*., 1988).

In terms of ectoparasite transmission, male reedbuck were more central than females. While mostly solitary, males move between females during the mating season, increasing contact levels with conspecifics. Junker *et al*. (2022) attributed higher centrality of males in nyala–helminth networks to their more varied social interactions. In addition, higher vagility and body mass have been associated with increased species richness and/or abundance in helminth and ectoparasite communities (Gallivan and Horak, 1997; Spickett *et al*., 2017*b*).

## Conclusion

Despite the fact that the sample size for age/sex categories was relatively small, resulting in a fairly low power to detect differences in prevalence or burden, some effects of host age and sex on the helminth and ectoparasite communities of reedbuck were found. However, the effect of locality on parasite communities exceeded that of host sex and age, especially, and as predicted, in ectoparasites. In addition, complex interactions between reedbuck traits, parasite traits and local environmental conditions seem to modulate the risk of infection and thus helminth and ectoparasite community and network structure varied between locations. This emphasizes our earlier findings in nyalas that pooling communities from different locations when investigating parasite community and network structure may skew results and obscure location-specific patterns.

While helminth communities of reedbuck were similar to those of nyalas in that host age played a larger role in structuring communities and networks than sex, host traits were less influential than in nyalas and the main differences in reedbuck–parasite interactions were locality dependent. We attribute this to the differences in sociality between the 2 host species. The mostly solitary, non-territorial lifestyle of reedbuck seems to negate some of the more pronounced age- and sex-dependent variation in behavioural patterns seen in gregarious nyalas.

## Supporting information

Junker et al. supplementary material 1 Junker et al. supplementary material

Junker et al. supplementary material 2 Junker et al. supplementary material

Junker et al. supplementary material 3 Junker et al. supplementary material

Junker et al. supplementary material 4 Junker et al. supplementary material

## Data Availability

All data generated or analysed during this study are included in this published article. The datasets used and/or analyses are available from the corresponding author upon reasonable request.

## Supplementary material

The supplementary material for this article can be found at https://doi.org/10.1017/S0031182024000532.
