# Supplementary material for: Ecto- and endoparasites of common reedbuck, Redunca arundinum, at 2 localities in KwaZulu-Natal Province, South Africa: community and network structure
Source: Parasitology. 2024 May 27;151(7):657–70. doi: 10.1017/S0031182024000532 (PMC11474021; doi:10.1017/S0031182024000532)
Supplement: Junker et al. supplementary material 1 — Junker et al. supplementary material [file S0031182024000532sup001.docx]

**Supplementary material. Table S1.** Prevalence and abundance (range; total count) of helminth parasites collected from common reedbuck, *Redunca arundinum* (Boddaert), at two localities in KwaZulu-Natal Province.

| Locality | Himeville (n = 26) | | | Eastern Shores Nature Reserve (n = 30) | | |
| --- | --- | --- | --- | --- | --- | --- |
| Parasite taxon | *N* | Prev. (%) | Range; total count | *N* | Prev. (%) | Range; total count |
| **Nematoda** |  |  |  |  |  |  |
| *Cooperia hungi* Mönnig, 1931 | - | - | - | 1 | 3.3 | 0–100; 100 |
| *Cooperia yoshidai* Mönnig, 1939 | 23 | 88.5 | 0–9179; 37969 | 24 | 80.0 | 0–24519; 109909 |
| *Cooperia yoshidai* L4 | 6 | 23.1 | 0–150; 317 | 13 | 43.3 | 0–17625; 22457 |
| *Cooperioides hepaticae* Ortlepp, 1938 | - | - | - | 1 | 3.3 | 0–5; 5 |
| *Cooperia*-like females | - | - | - | 2 | 6.7 | 0–6733; 9209 |
| *Cooperia*-like L4 | - | - | - | 2 | 6.7 | 0–51; 101 |
| *Dictyocaulus* *viviparus* Railliet & Henri, 1907 | 14 | 53.8 | 0–68; 203 | 24 | 80.0 | 0–297; 1628 |
| *Gaigeria* sp. | 2 | 7.7 | 0–25; 50 | 1 | 3.3 | 0–10; 10 |
| *Gongylonema* sp. | - | - | - | 4 | 13.3 | 0–7; 16 |
| *Haemonchus contortus* (Rudolphi, 1803) | 11 | 42.3 | 0–201; 1080 | 28 | 93.3 | 0–2726; 18937 |
| *Haemonchus contortus* L4 | 5 | 19.2 | 0–308; 575 | 22 | 73.3 | 0–3566; 15459 |
| *Impalaia* *tuberculata* Mönnig, 1923 | - | - | - | 2 | 6.7 | 0–250; 251 |
| *Longistrongylus schrenki* Ortlepp, 1939 | 17 | 65.4 | 0–650; 2065 | 28 | 93.3 | 0–1367; 8979 |
| *Longistrongylus* *schrenki* L4 | - | - | - | 18 | 60.0 | 0–664; 4684 |
| *Oesophagostomum* *columbianum* Curtice, 1890 | - | - | - | 3 | 10.0 | 0–50; 76 |
| *Oesophagostomum* *comlumbianum* L4 | - | - | - | 3 | 10.0 | 0–26; 29 |
| *Ostertagia* *ostertagi* (Stiles, 1892) | 3 | 11.5 | 0–50; 101 | - | - | - |
| *Setaria* *bicoronata* (von Linstow, 1901) | 14 | 53.8 | 0–11; 54 | 17 | 56.7 | 0–33; 207 |
| *Setaria* *labiatopapillosa*  (Alessandrini, 1848) | 1 | 3.8 | 0–1; 1 | - | - | - |
| *Setaria* sp. females | - | - | - | 6 | 20.0 | 0–28; 89 |
| *Skrjabinema* sp. | - | - | - | 11 | 36.7 | 0–11081; 37305 |
| *Trichostrongylus* *falculatus* Ransom, 1911 | 4 | 15.4 | 0–25; 78 | - | - | - |
| *Trichostrongylus* *falculatus* L4 | 1 | 3.8 | 0–3; 3 | - | - | - |
| *Trichuris* sp. females | 1 | 3.8 | 0–25; 25 | 1 | 3.3 | 0–25; 25 |
| **Trematoda** |  |  |  |  |  |  |
| Paramphistominae | 17 | 65.4 | 0–251; 1494 | 1 | 3.3 | 0–183; 183 |
| **Cestoda** |  |  |  |  |  |  |
| *Moniezia* *benedeni* (Moniez, 1879) | 1 | 3.8 | 0–1; 1 | 1 | 3.3 | 0–1; 1 |
| *Taenia* *hydatigena* Pallas, 1766 metacestodes | 3 | 11.5 | 0–1; 3 | - | - | - |

Prev. – prevalence. L4 – Fourth stage larvae.
