# Supplementary material for: Ecto- and endoparasites of common reedbuck, Redunca arundinum, at 2 localities in KwaZulu-Natal Province, South Africa: community and network structure
Source: Parasitology. 2024 May 27;151(7):657–70. doi: 10.1017/S0031182024000532 (PMC11474021; doi:10.1017/S0031182024000532)
Supplement: Junker et al. supplementary material 2 — Junker et al. supplementary material [file S0031182024000532sup002.docx]

**Supplementary material. Table S2.** Prevalence and abundance (range; total count) of ectoparasites collected from common reedbuck, *Redunca arundinum* (Boddaert), at two localities in KwaZulu-Natal Province.

| Locality | Himeville (n = 25) | | | Eastern Shores Nature Reserve (n = 26) | | |
| --- | --- | --- | --- | --- | --- | --- |
| Parasite taxon | *N* | Prev. (%) | Range; total count | *N* | Prev. (%) | Range; total count |
| **Adult ticks** |  |  |  |  |  |  |
| *Amblyomma hebraeum* Koch, 1844 | - | - | - | - | - | - |
| *Amblyomma marmoreum* Koch, 1844 | - | - | - | - | - | - |
| *Haemaphysalis* sp*.* | - | - | - | - | - | - |
| *Ixodes* sp*.* | 1 | 4.0 | 0–2; 2 | - | - | - |
| *Rhipicephalus appendiculatus* Neumann, 1901 | - | - | - | 5 | 19.2 | 0–148; 266 |
| *Rhipicephalus decoloratus* (Koch, 1844) | - | - | - | 2 | 7.7 | 0–2; 4 |
| *Rhipicephalus evertsi evertsi* Neumann, 1897 | 1 | 4.0 | 0–2; 2 | 3 | 11.5 | 0–4; 8 |
| *Rhipicephalus lounsburyi* Walker, 1990 | 2 | 8.0 | 0–18; 26 | - | - | - |
| *Rhipicephalus maculatus* Neumann, 1901 | - | - | - | - | - | - |
| *Rhipicephalus muehlensi* Zumpt, 1943 | - | - | - | 2 | 7.7 | 0–2; 4 |
| *Rhipicephalus* sp*.* | - | - | - | - | - | - |
| **Immature ticks** |  |  |  |  |  |  |
| *Amblyomma hebraeum* Koch, 1844 | - | - | - | 5 | 19.2 | 0–34; 48 |
| *Amblyomma marmoreum* Koch, 1844 | - | - | - | 3 | 11.5 | 0–18; 30 |
| *Haemaphysalis* sp*.* | - | - | - | 4 | 15.4 | 0–50; 62 |
| *Ixodes* sp*.* | 9 | 36.0 | 0–50; 118 | - | - | - |
| *Rhipicephalus appendiculatus* Neumann, 1901 | 2 | 8.0 | 0–2; 4 | 15 | 57.7 | 0–3788; 6746 |
| *Rhipicephalus decoloratus* (Koch, 1844) | 9 | 36.0 | 0–18; 44 | 11 | 42.3 | 0–110; 280 |
| *Rhipicephalus evertsi evertsi* Neumann, 1897 | 22 | 88.0 | 0–240; 1274 | 22 | 84.6 | 0–735; 1721 |
| *Rhipicephalus lounsburyi* Walker, 1990 | - | - | - | - | - | - |
| *Rhipicephalus maculatus* Neumann, 1901 | - | - | - | 3 | 11.5 | 0–148; 250 |
| *Rhipicephalus muehlensi* Zumpt, 1943 | - | - | - | 9 | 34.6 | 0–58; 156 |
| *Rhipicephalus* sp*.* | - | - | - | 3 | 11.5 | 0–530; 978 |
| **Adult lice** |  |  |  |  |  |  |
| *Damalinia reduncae* Bedford, 1929 | 22 | 88.0 | 0–2522; 4553 | 8 | 30.8 | 0–96; 184 |
| *Linognathus fahrenholzi* Paine, 1914 | 12 | 48.0 | 0–512; 584 | 5 | 19.2 | 0–8; 25 |
| **Immature lice** |  |  |  |  |  |  |
| *Damalinia reduncae* Bedford, 1929 | 15 | 60.0 | 0–946; 3515 | 8 | 30.8 | 0–54; 86 |
| *Linognathus fahrenholzi* Paine, 1914 | 11 | 44 | 0–204; 242 | 5 | 19.2 | 0–32; 48 |

Prev. – prevalence.
