# Supplementary material for: Ecto- and endoparasites of common reedbuck, Redunca arundinum, at 2 localities in KwaZulu-Natal Province, South Africa: community and network structure
Source: Parasitology. 2024 May 27;151(7):657–70. doi: 10.1017/S0031182024000532 (PMC11474021; doi:10.1017/S0031182024000532)
Supplement: Junker et al. supplementary material 3 — Junker et al. supplementary material [file S0031182024000532sup003.docx]

**Supplementary material. Table S3.** Phylogenetic and life cycle traits of helminth parasites collected from common reedbuck, *Redunca arundinum* (Boddaert), at two localities in KwaZulu-Natal Province, South Africa.

| **Parasite taxon** | **Family** | **Taxon code** | **Site in host** | **Life cycle** | **Transmission mode** |
| --- | --- | --- | --- | --- | --- |
| **Phylum: Nematoda** |  |  |  |  |  |
| **Class: Chromadorea** |  |  |  |  |  |
| **Order: Strongylida** |  |  |  |  |  |
| *Gaigeria* sp. | Ancylostomatidae | Gaispe | Small intestine | Direct | Percutaneous invasion |
| *Dictyocaulus* *viviparus* Railliet & Henri, 1907 | Dictyocaulidae | Dicviv | Lungs | Direct | Trophic: accidental ingestion of L3 |
| *Oesophagostomum* *coumbianum* Curtice, 1890 | Chabertiidae | Oescol | Large intestine | Direct | Trophic: accidental ingestion of L3 |
| *Cooperia* *hungi* Mönnig, 1931 | Cooperiidae | Coohun | Small intestine | Direct | Trophic: accidental ingestion of L3 |
| *Cooperia* *yoshidai* Mönnig, 1939 | Cooperiidae | Cooyos | Small intestine | Direct | Trophic: accidental ingestion of L3 |
| *Cooperia*-like females | Cooperiidae | Coopli | Small intestine | Direct | Trophic: accidental ingestion of L3 |
| *Cooperioides* *hepaticae* Ortlepp, 1938 | Cooperiidae | Coohep | Liver, bile ducts | Direct | Trophic: accidental ingestion of L3 |
| *Impalaia* *tuberculata* Mönnig, 1923 | Cooperiidae | Imptub | Small intestine | Direct | Trophic: accidental ingestion of L3 |
| *Haemonchus* *contortus* (Rudolphi, 1803) | Haemonchidae | Haecon | Abomasum | Direct | Trophic: accidental ingestion of L3 |
| *Longistrongyus* *schrenki* Ortlepp, 1939 | Haemonchidae | Lonsch | Abomasum | Direct | Trophic: accidental ingestion of L3 |
| *Ostertagia* *ostertagi* (Stiles, 1892) | Haemonchidae | Ostost | Abomasum | Direct | Trophic: accidental ingestion of L3 |
| *Trichostrongylus* *falculatus* Ransom, 1911 | Trichostrongylidae | Trifal | Small intestine | Direct | Trophic: accidental ingestion of L3 |
| **Order: Oxyurida** |  |  |  |  |  |
| *Skrjabinema* sp. | Oxyuridae | Skrspe | Large intestine | Direct | Trophic: accidental ingestion of eggs containing L3 |
| **Order: Spirurida** |  |  |  |  |  |
| *Gongylonema* sp. | Gongylonematidae | Gonspe | Oesophagus | Indirect | Trophic: accidental ingestion of intermediate host |
| *Setaria* *bicoronata* (von Linstow, 1901) | Onchocercidae | Setbic | Abdominal cavity | Indirect | Vector transmitted |
| *Setaria* *labiatopapillosa* (Alessandrini, 1848) | Onchocercidae | Setlab | Abdominal cavity | Indirect | Vector transmitted |
| *Setaria* sp. females | Onchochercidae | Setspe | Abdominal cavity | Indirect | Vector transmitted |
| **Class: Enoplea** |  |  |  |  |  |
| **Order: Trichinellida** |  |  |  |  |  |
| *Trichuris* sp. females | Trichuridae | Truspe | Large intestine | Direct | Trophic: accidental ingestion of eggs containing L1 |
| **Phylum: Platyhelminthes** |  |  |  |  |  |
| **Class: Trematoda** |  |  |  |  |  |
| **Superfamily: Paramphistomoidea** |  |  |  |  |  |
| Paramphistominae | Paramphistomidae | Param | Rumen | Indirect | Percutaneous invasion of intermediate host; followed by accidental ingestion of intermediate host by definitive host |
| **Class: Cestoda** |  |  |  |  |  |
| **Order: Cyclophyllidea** |  |  |  |  |  |
| *Moniezia* *benedeni* (Moniez, 1879) | Anoplocephalidae | Monben | Small intestine | Indirect | Trophic: accidental ingestion of intermediate host |
| *Taenia* *hydatigena* Pallas, 1766 metacestodes | Taeniidae | Taehyd | Metacestodes in the musculature | Indirect | Trophic: predator-prey relationship between definitive and intermediate host |

L1 – first-stage larva; L3 – third-stage larva
