# Supplementary material for: Ecto- and endoparasites of common reedbuck, Redunca arundinum, at 2 localities in KwaZulu-Natal Province, South Africa: community and network structure
Source: Parasitology. 2024 May 27;151(7):657–70. doi: 10.1017/S0031182024000532 (PMC11474021; doi:10.1017/S0031182024000532)
Supplement: Junker et al. supplementary material 4 — Junker et al. supplementary material [file S0031182024000532sup004.docx]

**Supplementary material. Table S4.** Phylogenetic and life cycle traits of ectoparasites collected from common reedbuck, *Redunca arundinum* (Boddaert), at two localities in KwaZulu-Natal Province, South Africa.

| **Parasite taxon** | **Family** | **Taxon code** | **Site on host (adults)** | **Adults and IM share host preference** | **Seasonality of adults** |
| --- | --- | --- | --- | --- | --- |
| **Phylum: Arthropoda** |  |  |  |  |  |
| **Class: Arachnida** |  |  |  |  |  |
| **Order: Ixodida** |  |  |  |  |  |
| *Amblyomma* *hebraeum* Koch, 1844 | Ixodidae | Ambheb | Underside: lower peri-anal region, udder, around genitalia and in the axillae | Yes | Summer |
| *Amblyomma* *marmoreum* Koch, 1844 | Ixodidae | Ambmar | Underside: soft skin around head, base of legs and tail | No | Spring to summer |
| *Haemaphysalis* sp. | Ixodidae | Haespe | - | - | - |
| *Ixodes* sp. | Ixodidae | Ixospe | - | - | - |
| *Rhipicephalus* *appendiculatus* Neumann, 1901 | Ixodidae | Rhiapp | Ear pinnae | Yes | Summer |
| *Rhipicephalus* *decoloratus* (Koch, 1844) | Ixodidae | Rhidec | Any part of the body | Yes | Spring |
| *Rhipicephalus* *evertsi* *evertsi* Neumann, 1897 | Ixodidae | Rhieve | Caudal region: hairless area around anus and groin | Yes | Summer |
| *Rhipicephalus* *lounsburyi* Walker, 1990 | Ixodidae | Rhilou | Underside: around feet | No | Winter |
| *Rhipicephalus* *maculatus* Neumann, 1901 | Ixodidae | Rhimac | Underside | Yes | Summer |
| *Rhipicephalus* *muehlensi* Zumpt, 1943 | Ixodidae | Rhimue | Ear pinnae | Yes | All year round |
| *Rhipicephalus* sp. | Ixodidae | Rhispe | - | - | - |
| **Class: Insecta** |  |  |  |  |  |
| **Order: Phthiraptera** |  |  |  |  |  |
| **Suborder: Ischnocera** |  |  |  |  |  |
| *Damalinia reduncae* Bedford, 1929 | Trichodectidae | Damred | Any part of the body | Yes | Cooler months |
| **Suborder: Anoplura** |  |  |  |  |  |
| *Linognathus fahrenholzi* Paine, 1914 | Lignognathidae | Linfah | Any part of the body | Yes | Cooler months |

IM – immatures
